# Influence of sagittal pelvic attitude on gait pattern in normally developed people and interactions with neurological pathologies: A pilot study

**DOI:** 10.3389/fnhum.2022.797282

**Published:** 2022-08-04

**Authors:** Martina Favetta, Alberto Romano, Susanna Summa, Alessandra Colazza, Silvia Minosse, Gessica Vasco, Enrico Castelli, Maurizio Petrarca

**Affiliations:** ^1^Department of Neurorehabilitation and Robotics, Movement Analysis and Robotics Laboratory (MARLab), “Bambino Gesù” Children’s Hospital, IRCCS, Rome, Italy; ^2^Department of Biomedicine and Prevention, University of Rome “Tor Vergata”, Rome, Italy

**Keywords:** gait deviations, biomechanics, gait mimicking, pelvic anteversion, pelvic posteroversion

## Abstract

**Background:**

Gait Analysis of healthy people, imitating pathological conditions while walking, has increased our understanding of biomechanical factors. The influence of the pelvis as a biomechanical constraint during gait is not specifically studied. How could mimicking a pelvic attitude influence the dynamic mechanical interaction of the body segments? We proposed an investigation of the pelvic attitude role on the gait pattern of typically developed people when they mimicked pelvic anteversion and posteroversion.

**Materials and methods:**

Seventeen healthy volunteers were enrolled in this study (mean age 24.4 ± 5.5). They simulated a pelvic anteversion and posteroversion during walking, exaggerating these postures as much as possible. 3D gait analysis was conducted using an optoelectronic system with eight cameras (Vicon MX, Oxford, United Kingdom) and two force plates (AMTI, Or-6, Watertown, MA, United States). The kinematic, kinetic, and spatio-temporal parameters were compared between the three walking conditions (anteversion, posteroversion, and normal gait).

**Results:**

In Pelvic Anteversion gait (PA) we found: increased hip flexion (*p* < 0.0001), increased knee flexion during stance (*p* = 0.02), and reduction of ankle flexion-extension Range of Motion (RoM) compared with Pelvic Normal gait (PN). In Pelvic Posteroversion gait (PP) compared with PN, we found: decreased hip flexion-extension RoM (*p* < 0.01) with a tendency to hip extension, decreased knee maximum extension in stance (*p* = 0.033), and increased ankle maximum dorsiflexion in stance (*p* = 0.002).

**Conclusion:**

The configuration of PA contains gait similarities and differences when compared with pathologic gait where there is an anteversion as seen in children with Cerebral Palsy (CP) or Duchenne Muscular Dystrophy (DMD). Similarly, attitudes of PP have been described in patients with Charcot-Marie-Tooth Syndrome (CMT) or patients who have undergone Pelvic Osteotomy (PO). Understanding the dynamic biomechanical constraints is essential to the assessment of pathological behavior. The central nervous system adapts motor behavior in interaction with body constraints and available resources.

## Introduction

The relationship between biomechanical constraints and neuromuscular systems in patients with cerebral palsy is not clear even if it is crucial knowledge in rehabilitation and surgery (Blumetti et al., 2019; Davids et al., 2019). In the simulated toe-walking, crouch, and crouch/equinus gait, Thomas et al. (1996) described a decrease in both walking velocity and stride length which correlated with the increase in the number of joints involved. This pattern is also seen in patients with Cerebral Palsy (CP) (Sutherland and Davids, 1993).

Romkes and Brunner (2007) highlighted that the Gait Analysis (GA) patterns at ankle level, in healthy subjects mimicking hemiplegic walking, are similar to those of children with hemiplegic CP. Drefus et al. (2016) underlined that the knee and ankle kinematics are linked, in the absence of hamstring spasticity or contracture. Furthermore, the study of Davids et al. (1999) showed that the decrease in walking velocity and length of stride as well as the increased pelvic anteversion are similar in voluntary and obligatory toe walking.

Holt et al. (2000) suggested that, in subjects with CP, the available dynamic resources determine the walking strategies constrained by biomechanical solutions. In this perspective, the relationship between central and peripheral resources is mutual and not discernible (Crenna, 1998). That is, it is possible that the pattern is determined by biomechanical constraints related to changes in the peripheral resources available, while muscle activities act on the fine tuning of the movement.

Romkes and Brunner (2007) described differences in EMG patterns of the rectus femoris muscle between the toe walkers and the mimicking controls, and therefore, this activity is considered an entirely pathological primary deviation. Although this muscle has a proximal insertion on the pelvis, the authors did not control the pelvis in their study and consequently, their conclusions concerning the role of the rectus femoris need more detailed study. Amori et al. (2015) reported that the pelvis can act as the reference frame to achieve body standing balance during 3D postural perturbation.

Other studies investigated the pelvic obliquity and rotational restriction using a robot walker. It altered gait dynamics reducing stride length, gait velocity, and increasing the percentage of the stance phase. Furthermore, the pelvic restrictions caused limited ankle plantarflexion at the terminal stance, knee flexion at mid-swing and hip extension at mid-stance, contributing to the reduction of RoMs in all of the joints of the lower limbs (Mun et al., 2016). Another study (Alingh et al., 2019) investigated the immediate after-effect of robot-assisted gait with pelvic support or pelvic rotational constraint on overground walking in healthy adults. These authors showed that robot-assisted gait training with pelvic constraint has an immediate negative after-effect on the overground walking pattern in healthy subjects, while the robot-assisted gait training with pelvic support better resembles the natural gait pattern. Therefore, it is known that a fixation of the pelvis severely affects gait dynamics.

The development of exoskeleton allowed the creation of systems of biomechanical constraints externally imposed. In any case, the limit of these studies is that the imposed constraints did not allow the simulation of the pathological condition nor investigated the pelvis tilt.

The influence of the pelvis tilt as a biomechanical constraint during gait using *in vivo* simulation is not specifically studied. In our study, we propose to extend the previously mentioned research objectives to include the role of pelvic tilt attitude in the gait pattern of normally developed people mimicking pelvic anteversion and posteroversion. Kinematic, kinetic, and spatio-temporal parameters were compared, using 3D gait analysis, to identify the adopted strategies in the biomechanical challenge. These observations could improve our understanding of the effect of biomechanics on pathologic gait evolution useful for rehabilitation and surgery decision making. In this study, we recruited young adults because we needed their collaboration to perform precise biomechanical simulations. The correct imitation of a gait pattern is difficult to obtain with younger children. Furthermore, the gait pattern generally stabilizes at around 7 years of age (Sutherland, 1997).

## Materials and methods

### Participants

Seventeen healthy volunteers were enrolled in this study (12 females, 5 males; mean age 24.4 ± 5.5, range 12-31; mean height 164.7 ± 11.9; mean mass 58.8 ± 11.8). Selection criteria included no prior cardiovascular history, neurological or musculoskeletal disorders that would influence gait. All subjects agreed to participate in the study. The study was conducted in respect of the Helsinki declaration.

### Experimental set up

**FIGURE 1:**
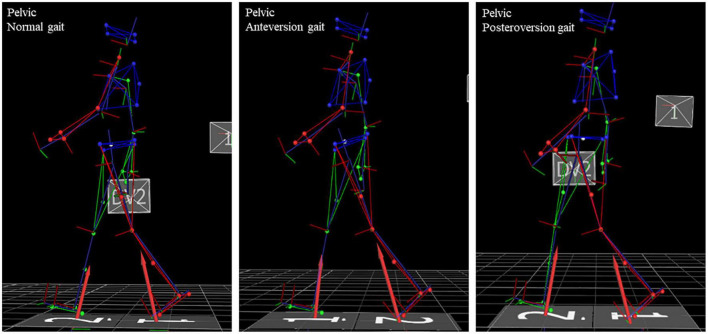
Full body reconstruction 3D gait analysis of the three conditions of experiment.

Seventeen healthy participants simulated a pelvic anteversion and posteroversion during walking, exaggerating the postures as much as possible. Each participant had their own time to learn and adapt. Data gathering started when they reached a stable behavior. All subjects were naïve to pathological gait characteristics. 3D gait analysis was conducted using an optoelectronic system with eight cameras (Vicon MX, Oxford, United Kingdom), two force plates (AMTI, Or-6, Watertown, MA, United States) hidden in the middle section of a 12-meter walkway and a synchronic video system to assist clinical interpretation of data. The sampling rate was set at 200 Hz for the motion capture system and at 1,000 Hz for the two force plates. The kinematic and kinetic full-body models were reconstructed using the plug-in gait protocol (Davis et al., 1991; Benedetti et al., 2017; Schirinzi et al., 2018). We asked three subjects to perform the experiment. An experienced clinician verified the feasibility and solidity of the responses before increasing the study sample. Participants were asked to walk barefoot at their natural cadence and self-selected comfortable speed. The pelvic angle was controlled ongoing using the real-time tilt plotting guaranteed by our 3D gait analysis system. The participants didn’t receive any feedback or further instructions. Only when the participants reached a stable behavior we became the acquisition. Ten trials were collected after two or more familiarization tests for each of the three conditions (natural gait and gait with pelvis in anteversion and in posteroversion in a random distribution between the participants). Participants performed the test without compensation of the trunk and the upper body as shown in Figure 1. The analysis involved kinematic, kinetic and spatio-temporal parameters. We looked at the sagittal, frontal, and transversal plane of each lower limb joint. We extracted clinical parameters: Range of Motion (RoM), mean, Initial Contact (IC), and peak values of the angle joints, moments and powers. Furthermore, we studied the foot progress angle (FPA), i.e., the angle of the foot in respect to the direction of progression. Kinematic and kinetic temporal series were normalized to the stride duration. Kinetic data were normalized to subject’s weight. Average values of three consistent trials were analyzed for each condition and each participant. The consistent trials were determined by expert operators with over 30 years of experience in clinical instrumented gait analysis. The trials were selected by watching videos and choosing the tests in which the subject was more natural in performing the motor task. All parameters were evaluated separately for the two limbs. Given the non-variability between the two body sides, we calculated the average between the two contests.

### Data analysis

The extraction of clinical parameters and the determination of the significant differences between the three conditions were performed. Shapiro-Wilk and Bartlett tests were used to verify, respectively, if the parameters were normally distributed and if the variance was uniform. If the assumptions of normality and of homogeneity of variances were verified, one-way ANOVA was performed. If these assumptions were not verified, we ran the Kruskal-Wallis test. The Bonferroni *post-hoc* analysis was applied to verify which pairs of groups differ significantly. A *p* < 0.05 was considered to indicate statistical significance.

## Results

**FIGURE 2:**
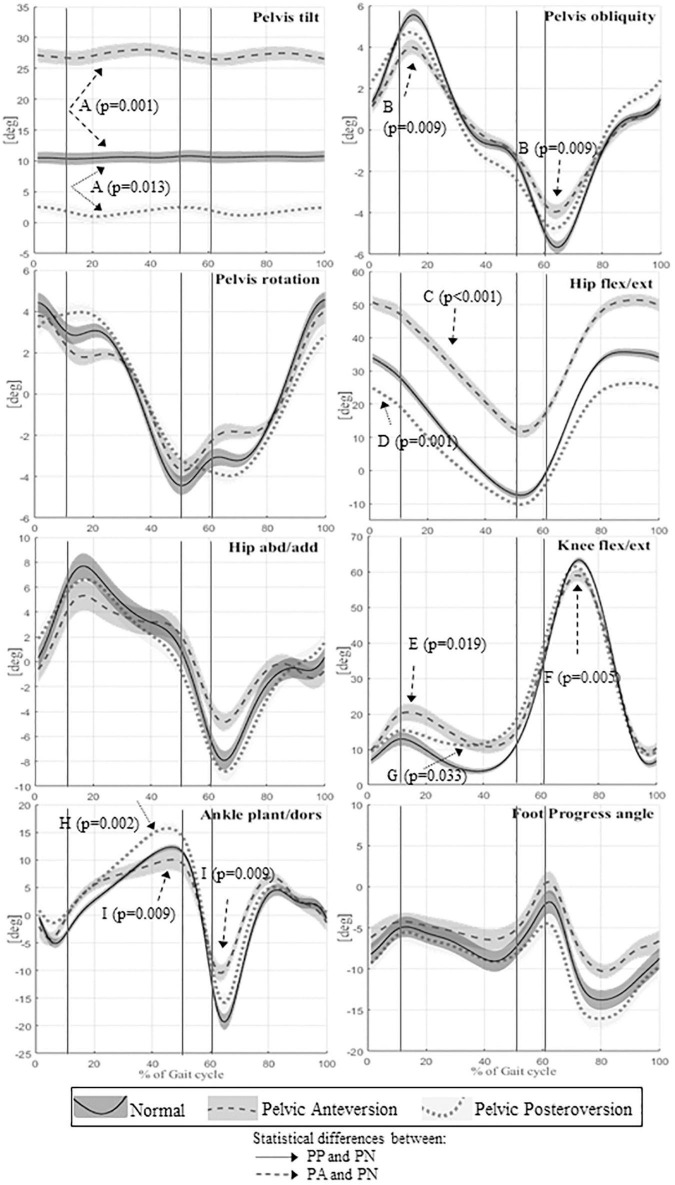
Means and standard deviation of kinematics angles of lower limb joint movements on the sagittal plane of the whole sample; the angles of the pelvic tilt, rotation and obliquity; the angles on frontal plane of hip and ankle. The angles of joint rotation in degrees are reported on the vertical axis, the time of the gait cycle in percentage is reported on the horizontal axis. Continuous lines represent averaged group value of Normal gait (PN), dashed lines represent averaged group value of Pelvic Anteversion gait (PA), dotted lines represent averaged group value of Pelvic Posteroversion gait (PP). The shaded areas denote the standard deviation (SD), dark gray, normal gray, and light gray stand respectively for Normal, Anteversion, and Posteroversion. The three lines separate the first double support phase (first line), the single support phase (second line), and the second double support phase (third line). Dashed arrows represent statistical differences between PA and PN, dotted arrows represent statistical differences between PP and PN. A (Pelvis tilt mean), B (Pelvis obliquity RoM), C (Hip flexion/extension mean), D (Hip flexion/extension IC), E (Knee max flexion in stance), F (Knee flexion/extension RoM), G (Knee max extension in stance), H (Ankle max dorsiflexion instance), I (Ankle flexion/extension RoM).

**FIGURE 3:**
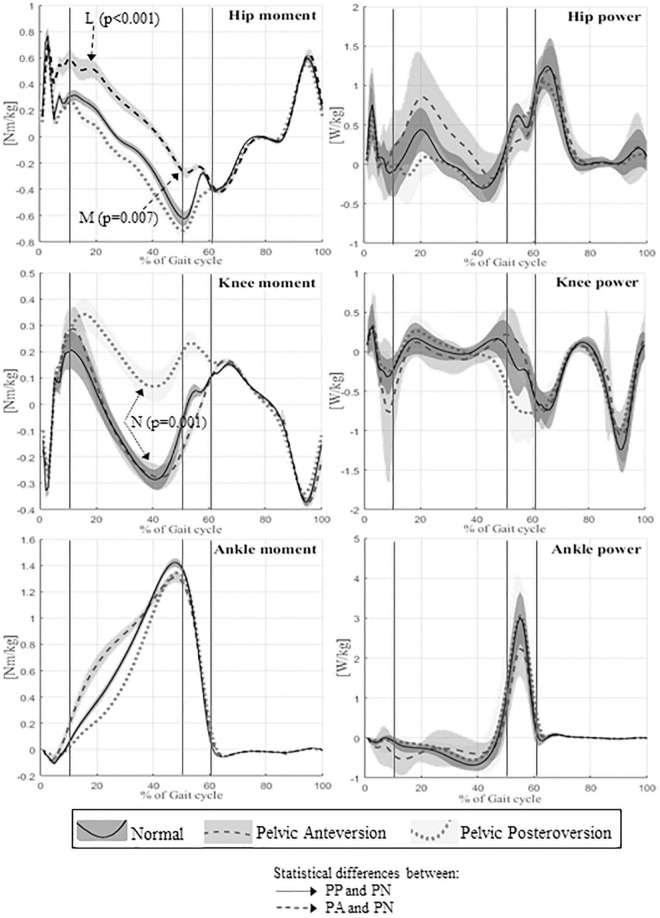
Means and standard deviation of kinetics of lower limbs joints of the whole sample (on the left panel: hip, knee, ankle moments; on the right panel: hip, knee, and ankle powers). The units of measure are reported on vertical axis, the time of gait cycle in percentage on the horizontal axis. Continuous lines represent averaged group value of Normal gait, dashed lines represent averaged group value of Pelvic Anteversion gait, and dotted lines represent averaged group value of Pelvic Posteroversion gait. The shaded areas denote the standard deviation (SD), dark gray, normal gray, and light gray stand respectively for Normal, Anteversion, and Posteroversion. The three lines separate the first double support phase (first line), the single support phase (second line), and the second double support phase (third line). Dashed arrows represent statistical differences between PA and PN, dotted arrows represent statistical differences between PP and PN. L (Hip maximum flexion moment), M (Hip max extension moment), N (Knee extension moment).

Descriptive statistics for all the spatio-temporal, kinematic, and kinetic parameters for the three different evaluations and comparisons between the three walking conditions are reported in Supplementary Table 1. Kinematic graphs are shown in Figure 2. Kinetic graphs are illustrated in Figure 3.

### Pelvic anteversion gait vs. pelvic normal gait

Walking in pelvic anteversion, subjects showed a significant decrease of the step length (*p* = 0.019), stride time (*p* = 0.042), and stride length (*p* = 0.014) compared with PN; there were no significant differences in walking velocity and double support time.

The kinematics of the lower limbs in PA showed a significant increase in the mean value of pelvic movement on the sagittal plane (*p* = 0.001) and a significant increase of pelvic tilt RoM (*p* < 0.0001). The pelvic obliquity RoM was reduced (*p* = 0.009). The hip showed a significant increase of flexion (*p* < 0.001) throughout the gait cycle. At knee level, we observed a reduction of flexion-extension RoM (*p* = 0.005) and increased maximum knee flexion during stance (*p* = 0.02). The ankle showed a decrease of flexion-extension RoM (*p* = 0.009) compared to PN. In PA, kinetic data showed a significant reduction of hip maximum extension moment (*p* = 0.007), an increase of hip maximum flexion moment (*p* < 0.001) and an increase of hip positive work (*p* = 0.008).

### Pelvic posteroversion gait vs. pelvic normal gait

Walking in pelvic posteroversion, subjects showed a significant decrease of velocity (*p* = 0.032), step length (*p* < 0.001), and stride length (*p* < 0.0001) compared with PN; there were no significant differences in double support time.

With respect to the kinematic lower limb values, in PP we found a significant increase of pelvic tilt RoM (*p* = 0.004) with reduction of the mean value (*p* = 0.013). At hip level, we observed a significant decrease of flexion at the initial contact (*p* = 0.001) and during the entire gait cycle (*p* < 0.0001), and a reduction of flexion-extension RoM (*p* < 0.01). The knee showed a significant decrease of the maximum extension in stance (*p* = 0.033), which is also anticipated (*p* = 0.002) compared to PN. At ankle level, we observed an increase of the maximum dorsiflexion in stance (*p* = 0.002). The kinetic data in PP showed a reduction of knee extension moment value (*p* = 0.001) and an increase of ankle positive work (*p* < 0.001; *p* < 0.05) compared with PN.

### Pelvic anteversion gait vs. pelvic posteroversion gait

For subjects simulating pelvic anteversion we observed: a decrease of the stance time percentage in the gait cycle (*p* = 0.025), a higher gait velocity (*p* = 0.033), and a decrease of the double support (*p* = 0.006) compared to PP. There were no significant changes in the step and stride length.

With respect to the kinematic lower limb values, in PA we found: an increase of the pelvic tilt mean (*p* < 0.0001), a decrease of pelvis obliquity at IC (*p* = 0.047), an increase of hip flexion during the gait cycle (*p* < 0.0001) and also at the IC (*p* < 0.0001), a reduction of hip abduction/adduction RoM (*p* < 0.001), an increase of hip maximum flexion (*p* < 0.0001), a decrease of hip maximum extension (*p* < 0.0001), and a decrease of maximum abduction (*p* = 0.01) compared with PP. There were no significant differences in knee kinematics between the two walking conditions. The ankle in PA showed a reduction of ankle flexion-extension RoM (*p* < 0.001) and a reduction of the ankle maximum dorsiflexion in stance (*p* < 0.01) compared to PP. Finally, PA is characterized by a greater intrarotation of foot progression angle (FPA) (*p* < 0.001), while in PP we observed a greater extrarotation. The kinetic data in PA showed a decrease of hip maximum extension moment in stance (*p* < 0.0001), an increase of hip maximum flexion moment (*p* < 0.001), an increase of hip positive work (*p* < 0.01), and a decrease of the ankle peak value of power generated (*p* < 0.01) with the reduction of ankle positive work (*p* < 0.01) compared to PP.

## Discussion

### Pelvic anteversion

The results reveal that the configuration of gait pattern induced by mimicking pelvic anteversion contains certain gait similarities and differences when compared with the pathological gait seen in children with Cerebral Palsy (Johnson et al., 1997; Bell et al., 2002; Petrarca et al., 2006; Armand et al., 2016) or with Duchenne Muscular Dystrophy (D’Angelo et al., 2009; Sienko Thomas et al., 2010; Goudriaan et al., 2018).

#### Cerebral palsy gait vs. pelvic anteversion gait

Similarities: shorter stride and step length (Johnson et al., 1997), increase of anterior pelvic tilt, increase of hip flexion, increase of knee flexion during stance (Armand et al., 2016), reduction of knee sagittal RoM, reduction of ankle sagittal RoM (Bell et al., 2002).

Differences: in healthy subjects mimicking gait with anteversion we did not find: reduction of gait velocity (Johnson et al., 1997), decrease of knee flexion during swing phase, loss of the first heel rocker at initial contact, increased plantarflexion in stance and swing (Bell et al., 2002; Armand et al., 2016). However, even if these parameters differ from those of subjects with CP, it is interesting to observe that they show a tendency, not statistically significant, toward the pathologic gait. Similarly, the PA shows a tendency to increased ankle dorsiflexion moment and to decreased ankle dorsiflexion in late stance.

#### Duchenne muscular dystrophy gait vs. pelvic anteversion gait

Similarities: decrease of walking speed, reduction of step and stride length, and more evident hip flexion in terminal swing (D’Angelo et al., 2009).

Differences: the gait of patients with DMD also presents increased knee extension in stance, absence of extensor knee moment (D’Angelo et al., 2009), increased knee flexion in swing. This variability of movement in the kinematics of the knee contributed to the increase in total knee range of motion (Goudriaan et al., 2018). There is an increase in ankle dorsiflexion during early stance, a decrease in dorsiflexion during late stance, and an increase in plantarflexion during swing. The kinetics of the DMD gait shows a lower power generation at the hip (Goudriaan et al., 2018).

### Pelvic posteroversion

Similarly, the gait pattern induced by mimicking pelvic posteroversion presented gait similarities and differences when compared with the gait of patients exhibiting posteroversion as seen in children with Charcot-Marie-Tooth Syndrome (Don et al., 2007; Newman et al., 2007; Ferrarin et al., 2012) or in children who have undergone pelvic osteotomy (Petrarca et al., 2014).

#### Charcot-marie-tooth gait vs. pelvic posteroversion gait

Similarities: lower gait velocity, decrease of step length, increased hip extension during stance, reduction of hip flexion during swing, increased knee flexion during stance, and greater ankle dorsiflexion during stance (Don et al., 2007). The increase in ankle dorsiflexion needs to be coordinated with a greater hip extension in order to preserve progression and balance.

Differences: CMT gait also presents an increase of hip RoM (Ferrarin et al., 2012), while an increase of the peak knee flexion in swing is only seen in some sub-clusters of CMT. Moreover, there is usually greater ankle plantar flexion at initial contact, a foot drop in swing phase (Ferrarin et al., 2012) and a low value of the ankle plantar flexion during final stance (Don et al., 2007); our sample however showed only a tendency of no statistical significance. Ankle power production is higher at mid-stance, and again intriguingly, our sample showed a tendency of no statistical significance. The hip extensor moment and power production during stance are increased (Ferrarin et al., 2012).

#### Pelvic osteotomy vs. pelvic posteroversion gait

Similarities: reduced gait velocity, reduced pelvic anteversion, increased knee flexion during stance phase, and reduction of knee extensor moment during stance (Petrarca et al., 2014).

Differences: reduced dorsal moment at the ankle (Petrarca et al., 2014), although, in our sample, it is only a tendency with no statistical significance.

### Assessing pathological behavior through dynamic biomechanical constraints

We can hypothesize that the similarities found between CP/PA-DMD/PA and CMT/PP-PO/PP are due to the biomechanical constraints of the body, while the differences are related to the pathological condition. Indeed, in CP excessive plantar flexion is caused by spasticity or deficits in selective motor control (Johnson et al., 1997; Crenna, 1998). In DMD the knee extension in stance compensates for the weakness of the quadriceps in order to improve balance and maintain body stability, the increased knee flexion in swing is required for the clearance of the foot to avoid tripping or falling due to excessive plantar flexion during swing (D’Angelo et al., 2009; Goudriaan et al., 2018). Muscle weakness of the dorsiflexors could cause the ankle kinematics described (Goudriaan et al., 2018). Weakness of the hip extensor could explain the lower power generation at the hip, resulting in a pelvic anterior tilt and a more flexed position of the hip (Goudriaan et al., 2018).

In CMT gait, the hip extensor moment and power production during stance are related to a greater activation of hip extensor muscles (Ferrarin et al., 2012). The mechanism used to prevent tripping is increased hip abduction and pelvic elevation at the swing time which prolongs activation of the gluteus medium muscle (Don et al., 2007).

Previous studies evaluated toe walker patients affected by CP and healthy subjects simulating toe walking (Davids et al., 1999; Romkes and Brunner, 2007; Rezgui et al., 2013). These researchers focused on examining descriptors of CP gait deviations that may be considered as primary or compensatory deviations.

The results of our study would seem to indicate an inseparable link between the kinematic gait deviations and the underlying neuromuscular pathology, which needs to be considered in a framework of continuous interactions. These interactions seem to induce a central nervous system reorganization producing new strategies during a process of searching for the best adaptive solution. The tools at our disposal do not allow us to distinguish the impact of damage to the nervous system from the contribution of biomechanical adaptations (Crenna, 1998; Holt et al., 2000). In addition to this limitation of our knowledge, there is also the lack of a universally accepted theoretical framework concerning bipedal gait (Vaughan, 2003).

This study could support the hypothesis that the pelvis plays a key role in linking the locomotor system and the upper body (Amori et al., 2015). Furthermore, it is also known that the pelvis attitude can play a role in the foot-terrain coupling, especially in pathological condition when the control on the foot is limited by the neurological lesion. In that case, a little adjustment in retroversion of the pelvis can compensate a reduced dorsiflexion of the foot allowing the recovery of the three rockers of the foot during the stance phase (Petrarca et al., 2011). Exploiting the reciprocal influence described, seems to face new clinical opportunities during assessment and training of the function. Changing its attitude in the sagittal plane, we modified the biomechanical configuration of the body, stopping the exploitation of the body’s degree of freedom in an interaction between the biomechanical configurations and the solutions of motor control. In fact, although the pathological conditions open a process of function redefinition, it is clear from this study that PA leads toward an increase of hip work and PP toward an increase of ankle work.

These two gait conditions showed some motor behaviors that resemble some characteristics of the pathological gait, even if they were not statistically significant. We speculated that specific configurations need time to emerge. Therefore, a possible limitation of this paper is the sudden collection of gait data, not giving the subject enough time to adapt to the new motor configuration; with time and training the tendency could become a new stable solution. Furthermore, these findings can be considered as preliminary data resulting from limited size of participants.

Finally, we can hypothesize that the pathological condition stops the full exploitation of the body’s degrees of freedom that contribute to limit the process of research of adaptive solutions. The process of recovering the function could reopen the exploitation of new adaptive solutions by including new biomechanical configurations in a personalized trial-and-error learning method.

## Data availability statement

The original contributions presented in this study are included in the article/Supplementary material, further inquiries can be directed to the corresponding author.

## Ethics statement

The studies involving human participants were reviewed and approved by Bambino Gesù Children’s Hospital Ethical Committee. The patients/participants provided their written informed consent to participate in this study.

## Author contributions

MF performed acquisition and interpretation of data, organized database, and wrote the first draft of the manuscript. AR contributed to acquisition and interpretation of data. SS performed data analysis and statistical analysis. AC contributed to acquisition of data. SM contributed to data analysis. GV and EC revised the manuscript critically for intellectual content. MP achieved conception and design of the study, interpretation of data, drafting the work, and review and editing. All authors contributed to manuscript revision, read, and approved the submitted version.
