# Supplementary material for: Influence of sagittal pelvic attitude on gait pattern in normally developed people and interactions with neurological pathologies: A pilot study
Source: Front Hum Neurosci. 2022 Aug 4;16:797282. doi: 10.3389/fnhum.2022.797282 (PMC9386486; doi:10.3389/fnhum.2022.797282)
Supplement: Supplementary file 1 [file Data_Sheet_1.PDF]

Supplementary Material

Table 1 Supplementary materials: spatio-temporal, kinematic and kinetic parameters. Values are averaged (std) per group. NS is "not significant". Statistical significance is indicated in bold type. % = percentage occurrence of the event along the gait cycle.

**Gait parameters**	**Conditions**			**P-Value**		
Spatio-temporal parameters	Pelvis Normal gait (PN)	Pelvis Anteversion gait (PA)	Pelvis Posteroversion gait (PP)	PA vs PN	PP vs PN	PA vs PP
%Stance	60,35 ± 1,08	60,12 ± 1,41	61,55 ± 1,95	NS	NS	**0,025**
Velocity (m/s)	1,14 ± 0,08	1,14 ± 0,13	1,04 ± 0,12	NS	**0,032**	**0,033**
Step Width (m)	0,18 ± 0,03	0,18 ± 0,04	0,19 ± 0,05	NS	NS	NS
Step Length (m)	0,62 ± 0,03	0,58 ± 0,04	0,56 ± 0,04	**0,019**	**0,0001**	NS
Stride Time (s)	1,09 ± 0,06	1,03 ± 0,07	1,09 ± 0,08	**0,042**	NS	NS
Stride Length (m)	1,25 ± 0,06	1,17 ± 0,08	1,13 ± 0,08	**0,014**	**< 0.0001**	NS
Double support (s)	0,12 ± 0,02	0,11 ± 0,02	0,13 ± 0,03	NS	NS	**0,006**
Stance Time (s)	0,66 ± 0,04	0,62 ± 0,05	0,67 ± 0,06	NS	NS	NS
Swing Time (s)	0,43 ± 0,02	0,41 ± 0,03	0,42 ± 0,03	NS	NS	NS
Kinematic parameters						
Pelvis tilt Mean	10,61 ± 3,83	27,18 ± 4,59	1,81 ± 4,72	**0,001**	**0,013**	**< 0,0001**
Pelvis tilt RoM	3,13 ± 0,82	5,49 ± 1,75	4,64 ± 1,30	**< 0,0001**	**0,004**	NS
Pelvis tilt IC	10,49 ± 3,98	27,15 ± 4,77	2,55 ± 4,48	**0,001**	**0,019**	NS
Pelvis obliquity Mean	-0,03 ± 0,11	0,03 ± 0,20	-0,00 ± 0,12	NS	NS	NS
Pelvis obliquity RoM	11,45 ± 2,41	8,57 ± 2,82	9,95 ± 2,81	**0,009**	NS	NS
Pelvis obliquity IC	1,34 ± 1,19	1,14 ± 1,40	2,37 ± 1,40	NS	NS	**0,047**
Pelvis rotation Mean	-0,01 ± 0,19	0,08 ± 0,31	-0,04 ± 0,33	NS	NS	NS
Pelvis rotation RoM	10,72 ± 3,14	9,74 ± 3,42	10,42 ± 3,29	NS	NS	NS
Pelvis rotation IC	4,42 ± 2,06	3,78 ± 2,26	3,21 ± 2,15	NS	NS	NS
Hip flexion/extension Mean	16,43 ± 4,73	34,84 ± 7,76	9,76 ± 6,04	**< 0,001**	NS	**< 0,0001**
Hip flexion/extension RoM	44,62 ± 4,04	42,45 ± 7,26	38,46 ± 5,04	NS	**0,007**	NS
Hip flexion/extension IC	33,92 ± 6,01	50,73 ± 8,26	24,90 ± 6,49	**< 0,0001**	**0,001**	**< 0,0001**
Hip abduction/adduction Mean	0,72 ± 3,47	0,85 ± 3,62	0,08 ± 3,77	NS	NS	NS
Hip abduction/adduction RoM	15,95 ± 2,78	11,64 ± 3,31	16,12 ± 3,45	**0,001**	NS	**< 0,001**
Hip abduction/adduction IC	0,32 ± 3,42	-0,62 ± 4,01	1,85 ± 4,08	NS	NS	NS
Hip max flexion	36,98 ± 4,89	53,82 ± 7,62	27,95 ± 5,91	**< 0,0001**	**< 0,0001**	**< 0,0001**
%Hip max flexion	74,22 ± 21,19	59,87 ± 22,77	66,76 ± 24,80	NS	NS	NS
Hip max extension	-7,64 ± 4,73	11,38 ± 7,90	-10,50 ± 5,89	**< 0,0001**	NS	**< 0,0001**
%Hip max extension	52,24 ± 1,23	53,14 ± 1,31	52,16 ± 1,57	NS	NS	NS
Hip max adduction	7,83 ± 4,31	6,03 ± 4,88	6,96 ± 4,85	NS	NS	NS
%Hip max adduction	16,39 ± 1,58	24,78 ± 10,97	18,03 ± 4,31	NS	NS	NS

Hip max abduction	-8,12 ± 3,18	-5,61 ± 3,07	-9,16 ± 3,60	NS	NS	**0,008**
%Hip max abduction	65,41 ± 1,38	63,10 ± 8,40	65,84 ± 2,05	NS	NS	Ns
Knee flexion/extension Mean	21,07 ± 3,60	24,64 ± 6,14	24,07 ± 4,77	NS	NS	NS
Knee flexion/extension RoM	61,16 ± 4,19	54,01 ± 7,71	56,83 ± 6,52	**0,005**	NS	NS
Knee flexion/extension IC	6,96 ± 4,81	9,83 ± 6,46	9,44 ± 5,45	NS	NS	NS
Knee max flexion in stance	15,29 ± 5,59	22,48 ± 9,37	19,49 ± 5,39	**0,019**	NS	NS
%Knee max flexion in stance	27,00 ± 16,62	23,26 ± 10,28	36,42 ± 15,30	NS	NS	NS
Knee max flexion in swing	63,72 ± 3,54	59,90 ± 7,03	62,56 ± 6,19	NS	NS	NS
%Knee max flexion in swing	73,26 ± 0,76	72,61 ± 1,56	71, 88 ± 1,57	NS	**0,007**	NS
Knee max extension in stance	2,84 ± 3,68	6,96 ± 5,60	7,40 ± 5,56	**0,063**	**0,033**	NS
% Knee max extension in stance	32,23 ± 9,17	21,52 ± 14,47	15,10 ± 13,96	**0,067**	**0,002**	NS
Ankle flexion/extension Mean	1,21 ± 2,74	2,94 ± 4,69	3,72 ± 2,96	NS	NS	NS
Ankle flexion/extension RoM	32,76 ± 6,00	26,12 ± 5,25	35,41 ± 7,15	**0,009**	NS	**< 0,001**
Ankle flexion/extension IC	-0,40 ± 3,61	-2,02 ± 8,33	0,89 ± 5,30	NS	NS	NS
Ankle max dorsiflexion in stance	12,61 ± 2,05	12,33 ± 5,27	17,20 ± 3,53	NS	**0,002**	**0,005**
%Ankle max dorsiflexion in stance	46,23 ± 1,99	40,69 ± 11,90	44,37	NS	NS	NS
Ankle max dorsiflexion in swing	5,28 ± 4.44	7,80 ± 4,05	5,28 ± 4,05	NS	NS	NS
%Ankle max dorsiflexion in swing	85,71 ± 3,60	83,47 ± 4,15	84,52 ± 4,82	**0,026**	NS	NS
Ankle max plantarflexion	2,56 ± 3,64	5,90 ± 5,17	5,72 ± 5,25	NS	NS	NS
%Ankle max plantarflexion	50,25 ± 20,87	60,71 ± 19,32	59,85 ± 22,82	NS	NS	NS
FPA rotation-Mean	-7,98 ± 4,77	-5,67 ± 4,36	-9,27 ± 5,00	NS	NS	NS
FPA rotation-RoM	15,44 ± 3,84	14,22 ± 4,12	16,87 ± 3,72	NS	NS	NS
FPA rotation-IC	-8,22 ± 5,32	-6,19 ± 5,53	-9,31 ± 5,05	NS	NS	NS
FPA max rotation	-0,72 ± 5,62	1,58 ± 5,55	-2,05 ± 5,25	NS	NS	NS
%FPA max rotation	58,18 ± 7,37	61,81 ± 6,58	51,56 ± 16,89	NS	NS	NS
FPA min rotation	-16,16 ± 4,18	-12,64 ± 3,80	-18,91 ± 5,07	NS	NS	**< 0,001**
%FPA min rotation	79,97 ± 6,48	75,03 ± 7,37	78,49 ± 8,56	NS	NS	NS
Kinetic parameters						
Hip max extension moment	-0,70 ± 0,14	-0,51 ± 0,13	-0,81 ± 0,22	**0,007**	NS	**< 0,0001**
%Hip min extension moment	46,49 ± 3,24	50,61 ± 3,62	46,74 ± 3,82	**0,016**	NS	**0,014**

Hip max flexion moment	0,43 ± 0,15	0,78 ± 0,30	0,40 ± 0,20	**< 0,001**	NS	**< 0,001**
%Hip max flexion moment	4,19 ± 2,49	4,81 ± 2,14	4,28 ± 2,47	NS	NS	NS
Hip max abduction moment	0,80 ± 0,18	0,77 ± 0,23	0,81 ± 0,19	NS	NS	NS
%Hip max abduction moment	23,33 ± 11,42	17,23 ± 9,87	24,28 ± 11,24	NS	NS	NS
HGenPSt	1,10 ± 0,23	1,32 ± 0,46	1,23 ± 0,30	NS	NS	NS
HAbsPSt	-0,44 ± 0,17	-0,58 ± 0,33	-0,54 ± 0,25	NS	NS	NS
HGenPSw	1,46 ± 0,31	1,45 ± 0,26	1,38 ± 0,35	NS	NS	NS
Hip positive work	0,13 ± 0,04	0,21 ± 0,08	0,13 ± 0,03	**0,008**	NS	**0,003**
Hip negative work	0,06 ± 0,03	0,05 ± 0,05	0,08 ± 0,05	NS	NS	NS
Knee min extension moment	-0,31 ± 0,16	-0,34 ± 0,18	-0,068 ± 0,19	NS	**0,001**	**< 0,001**
%Knee min extension moment	33,33 ± 4,35	32,03 ± 8,67	32,09 ± 14,46	NS	NS	NS
Knee max flexion moment	0,36 ± 0,17	0,40 ± 0,26	0,46 ± 0,13	NS	NS	NS
%Knee max flexion moment	23,89 ± 20,02	18,12 ± 14,86	27,32 ± 12,62	NS	NS	NS
Knee max power in stance	0,40 ± 0,17	0,51 ± 0,22	0,38 ± 0,21	NS	NS	NS
Knee min power in stance	-0,88 ± 0,19	-1,15 ± 0,71	-1,22 ± 0,36	NS	NS	NS
Knee max power in stance	0,25 ± 0,13	0,71 ± 0,84	0,20 ± 0,09	NS	NS	**0,042**
Knee positive work	0,05 ± 0,02	0,05 ± 0,04	0,05 ± 0,03	NS	NS	NS
Knee negative work	0,07 ± 0,02	0,10 ± 0,04	0,14 ± 0,06	NS	**< 0,001**	NS
Ankle min plantarflexion moment	-0,12 ± 0,06	-0,14 ± 0,08	-0,1 ± 0,05	NS	NS	NS
%Ankle min plantarflexion moment	4,51 ± 0,94	3,47 ± 2,05	5,43 ± 2,05	NS	NS	**0,007**
Ankle max dorsiflexion moment	1,43 ± 0,14	1,37 ± 0,13	1,40 ± 0,16	NS	NS	NS
%Ankle max dorsiflexion moment	47,44 ± 1,27	43,13 ± 10,26	47,59 ± 3,30	NS	NS	NS
Ankle max power	3,33 ± 0,64	2,79 ± 0,62	3,75 ± 1,13	NS	NS	**0,009**
Ankle min power	-0,81 ± 0,13	-1,18 ± 0,79	-0,85 ± 0,30	NS	NS	NS
Ankle positive work	0,26 ± 0,05	0,23 ± 0,06	0,31 ± 0,08	NS	**0,046**	**0,001**
Ankle negative work	0,17 ±0,03	0,17 ± 0,06	0,14 ± 0,05	NS	NS	NS
